# Current Trends in Biomaterial Utilization for Cardiopulmonary System Regeneration

**DOI:** 10.1155/2018/3123961

**Published:** 2018-04-29

**Authors:** Adegbenro Omotuyi John Fakoya, David Adeiza Otohinoyi, Joshua Yusuf

**Affiliations:** ^1^Department of Anatomical Sciences, All Saints University School of Medicine, Roseau, Dominica; ^2^All Saints University School of Medicine, Roseau, Dominica; ^3^All Saints University School of Medicine, Kingstown, Saint Vincent and the Grenadines

## Abstract

The cardiopulmonary system is made up of the heart and the lungs, with the core function of one complementing the other. The unimpeded and optimal cycling of blood between these two systems is pivotal to the overall function of the entire human body. Although the function of the cardiopulmonary system appears uncomplicated, the tissues that make up this system are undoubtedly complex. Hence, damage to this system is undesirable as its capacity to self-regenerate is quite limited. The surge in the incidence and prevalence of cardiopulmonary diseases has reached a critical state for a top-notch response as it currently tops the mortality table. Several therapies currently being utilized can only sustain chronically ailing patients for a short period while they are awaiting a possible transplant, which is also not devoid of complications. Regenerative therapeutic techniques now appear to be a potential approach to solve this conundrum posed by these poorly self-regenerating tissues. Stem cell therapy alone appears not to be sufficient to provide the desired tissue regeneration and hence the drive for biomaterials that can support its transplantation and translation, providing not only physical support to seeded cells but also chemical and physiological cues to the cells to facilitate tissue regeneration. The cardiac and pulmonary systems, although literarily seen as just being functionally and spatially cooperative, as shown by their diverse and dissimilar adult cellular and tissue composition has been proven to share some common embryological codevelopment. However, necessitating their consideration for separate review is the immense adult architectural difference in these systems. This review also looks at details on new biological and synthetic biomaterials, tissue engineering, nanotechnology, and organ decellularization for cardiopulmonary regenerative therapies.

## 1. Introduction

Cardiopulmonary disease refers to diverse forms of diseases affecting the heart and lungs. Some of these diseases might result in considerable damage to the tissues of these organs and occasionally might cause irreparable damage to parts of these organs, thus impairing their overall function, consequently resulting in the reduction in the quality of life of the affected individual. The duties of these two systems are so integral, such that a chronically diseased state in one will invariably affect the efficient functioning of the other [1].

Stem cells have been explored in regenerative therapies of both the heart and the lungs, and sections below will briefly consider this. However, the survival of these cells is largely dependent on the environment in which they are placed [2], hence the search for the suitable biomaterials that can potentiate survival, proliferation, differentiation, and engraftment of the transplanted cells to enhance tissue regeneration. Biomaterial scaffolds should provide not only physical support but also the chemical and biological clues needed in forming functional tissues in either the heart or the lungs [3].

In this review, we shall distinctly be considering the biomaterials that have been used in heart and pulmonary regenerative therapies. Also, this review will reveal a skew towards cardiovascular research over pulmonary research. This is an anticipated skew as the cardiovascular system occupies a critical central role in the overall functioning of the body. Thus, the restoration of a healthy heart will translate into increased quality of life universally, reducing morbidity and mortality. This fundamental knowledge is the driver for more research into possible ways of restoring structure and function to a damaged heart which is at immense risk by modern-day lifestyle.

## 2. Biomaterials for Cardiac Regeneration

The need for new therapeutic inventions for cardiovascular diseases (CVDs) has been consistently indicated by the increased rate of associated diseases [1]. Statistics estimate a total annual expense of 1.2 trillion US dollars by 2030 in the United States if the current therapeutic interventions for CVD are maintained [2]. Among various CVDs, the most common is myocardial infarction (MI), which is the leading cause of morbidity and death in developing and developed nations [4]. MI involves the pathogenesis of anaerobic respiration, the accumulation of reactive oxygen species, and the death of cardiomyocytes (CM), thus affecting the normal physiological process of the heart [5]. Post myocardial infarction, the CM extracellular matrix (ECM) undergoes inflammatory, proliferation, and maturation stages of tissue remodeling to support other healthy CM [6, 7]. However, the scar tissue or collagen formed by the remodeling of the ECM at the maturation stage does not participate in the concomitant beating of the heart due to loss of organized architecture [8], which eventually leads to cardiomegaly and, ultimately, heart failure [6]. Present-day remedies like surgical, pharmacological, and endovascular interventions only have soothing purposes and do not address the fundamental flaw, which is the loss of functional CM [9]. Though heart transplant remains effective, the availability of donors and the occurrence of immune rejection pose a serious disadvantage. The recent discovery of cardiomyogenesis in humans has brought to light the role of cardiac regeneration from stem cells [10]. Cell varieties such as embryonic stem cells, cardiac stem cells, endothelial progenitor cells, skeletal myoblasts, and bone marrow mononuclear cells have been recognized to have regenerative properties in cardiomyogenesis. The use of these stem cells still has drawbacks like poor cell delivery and integration, low survival rate, and long-term toxicity; however, it is believed that modified biomaterials could limit these hindrances [11].

CMs were previously thought to be postmitotic, but recent studies have shown that cardiac tissue possesses some intrinsic mitotic activity with some regenerative potential as it contains a diversity of stem cells with regenerative potential in various niches in the heart [12]. These cardiac stem cells (CSCs) can be categorized into the following groups: ckit cells [13], Sca1^+^ cells [14], IsI1^+^ cells [15], cardiosphere-derived cells (ckit^+^/Sca1^+^/flh1^+^) [16], cardiac mesoangioblasts [17], side population cells (expressing Abcg2/Mdr1) [18], and epicardial progenitors [19]. However, these cells alone are unable to regenerate the heart in the face of an MI, hence necessitating collaborative research into other solutions such as the use of biomaterials. Before an ideal biomaterial can be developed for suitable stem cell integration, a good knowledge of the heart's extracellular matrix (ECM) is required. Below, we briefly consider the cardiac ECM.

### 2.1. The Extracellular Matrix of the Heart

The cardiac ECM is a complex mesh of structural and nonstructural components for support and good cellular remodeling [20]. The structural component of the cardiac ECM consists mainly of cardiofibroblasts (Cfs) and collagen fibrils while the nonstructural element is made up of glycosylated proteins such as glycoproteins (GPs), glycosaminoglycan (GAG), and proteoglycans (PGs) [20, 21]. Other ECM components include cytokines and enzymes with their inhibitory factors [22]. Receptors and signaling proteins also serve as a vital makeup of the ECM.

During cardiac injury, cardiac fibroblasts (Cf) play a critical role in tissue repair and remodeling, during which they usually undergo phenotypic modulation to become myofibroblasts after TGF*β*1 and fibronectin variant activation. In tissue repair, Cf function to synthesize the ECM components such as collagen and alpha-smooth muscle actin among other components [22].

**Figure 1:**
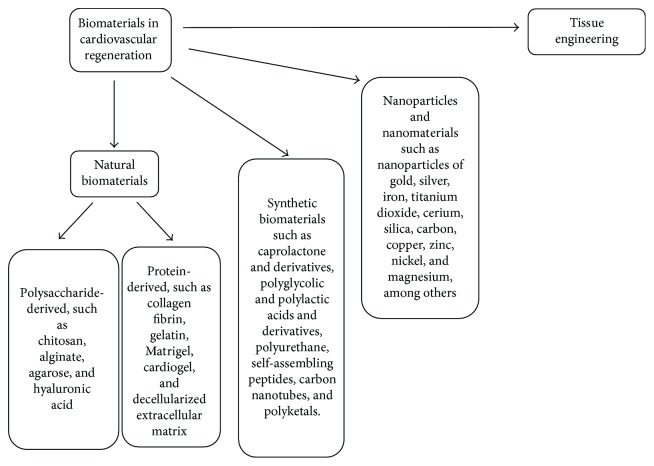
Classification of current biomaterials in cardiovascular regeneration.

Biomaterials in cardiomyogenesis are designed to model the natural cardiac ECM without its setbacks [22]. The healing process associated with MI involves the replacement of myocytes with granulation tissue despite the presence of CSCs within the heart tissue [1]. This occurs due to inadequate differentiation potential, not enough CSCs, and poor stimulation of stem cells within the myocardium, thus restricting cardiomyogenesis to the borders of the infarction, which is shown by maintained perfusion and signaling of CSCs by factors in the extracellular matrix around the borders of the infarct [1, 23]. It is stipulated that the introduction of stem cells with differentiation and growth signals (or biomaterials) could solve the setback in cardiomyogenesis [24]. Biomaterials, which are medical tools for grafting stem cells, are typically expected to be biodegradable and biocompatible, have minimal autoimmune stimulation, and possess a long half-life with sufficient reservoir capacity for bioactive molecules [11, 24]; however, when administered alone, biomaterials have a temporary remedy [23]. Biomaterials recognized for cardiomyogenesis can be broadly classified as either nature-based or synthetic; however, there are other categories that can serve as biomaterials in cardiovascular regeneration as discussed in this review (Figure 1).

### 2.2. Natural Biomaterials

Natural polymers are biodegradable matrices consisting of complex components found in native tissues. They can be easily manipulated, thus increasing or decreasing their half-life in vivo [25]. Natural biomaterials could either be protein, polysaccharide, or decellularized tissue-derived [26]. The ability of natural polymers to be biodegradable, biocompatible, and remoldable gives them an advantage over the synthetic polymers [24]. Protein and polysaccharide-based biomaterials are made by treating organic components with solvents and enzymes till interested components are separated from their biological source, whereas decellularized tissues involve the exclusion of cells from organic tissue, thereby maintaining the architectural and structural composition of the tissue [26].

#### 2.2.1. Polysaccharide-Derived Biomaterials in Cardiomyogenesis

Polysaccharides are described as polymeric carbohydrate molecules that are made up of monosaccharide units linked by glycosidic bonds [27]. The use of polysaccharides such as chitosan, alginate, agarose, and hyaluronic acid has been indicated in cardiomyogenesis [28, 29].

*(1) Chitosan*. Chitosan is the partial alkaline deacetylation of chitin, which is the second largest natural polymer after cellulose, and is readily found in the exoskeleton of insects and fungi [27]. Chitosan is a linear copolymer of *β*-(1–4)-D-glucosamine (deacetylated unit) and N-acetyl-D-glucosamine (acetylated unit) [27]. Foster et al. suggested that deacetylation should be above 75% to ensure optimal stem cell activity [28]. Chitosan supports cardiomyogenesis as it is biodegradable, biocompatible, marginally immunogenic, hydrophilic, hemostatic, nontoxic, and cohesive in character [30, 31]. Chitosan is usually combined with other composites to form complexes via electrostatic force or physical/chemical cross-linking due to its poor stability and low electrical conduction when used alone [30]. Studies done by Martins et al. showed that the coupling of carbon nanofibers with porous chitosan scaffolds improved the growth of neonatal rat heart cells in vitro. The supposed reason was that carbon nanofibers boosted electrical signaling transmissions between the cells [32]. Chitosan has also been observed to boost silk fibroin (SF) potential by improving the differentiation of rat mesenchymal stem cells (MSC) to CM in vitro, thus making the hybrid of chitosan and SF indicated in cardiomyogenesis [29]. Liu et al. also suggested that adipose-derived stem cells (ADSC) on chitosan enhanced the formation of transplantable spheroids with cardiac markers such as Gata4, Nkx2-5, Myh6, and Tnnt2 due to increased calcium signaling [33]. Thermosensitive conductive hydrogel generated from chitosan was also observed to support cardiomyogenic differentiation of MSC in the presence of gold nanoparticles (AuNPs) [34]. Hydrogels synthesized from chitosan also showed good potential in cardiomyogenesis after having been made electrically conductive by aniline oligomers or paratoluenesulfonate electrodeposition [35–37]. When tested for the effect of chitosan on the differentiation of brown adipose-derived stem cell (BASC) to CM, the expression of cardiac markers such as GATA-4, Nkx2.5, Myl7, Myh6, cTnI, and Cacna1a was noted [38]. The behavior of chitosan in vivo has also been monitored, with reports showing that BASC on chitosan increased cardiac function, neovascularization, and left ventricular pressure and reduced infarct size in rat models [39]. Chi et al. also reported that using cardiac patches consisting mainly of chitosan and without stem cells on myocardial infarcted rats led to increased wall thickness and reduced left ventricular dilation. However, there was no significant increase in neovascularization [39]. Simultaneous injection of the basic fibroblast growth factor (bFGF) with a temperature-sensitive chitosan hydrogel also enhanced the role of bFGF on neovascularization and cardiac function [40]. A common factor among researchers concerning the use of chitosan in cardiomyogenesis is in improving the potential of chitosan by measuring the synergistic effect combined with other factors, thus improving the integration of stem cells into cardiac tissues. This is because chitosan has a low mechanical resistance and is also susceptible to proteolytic enzymes when poorly acetylated [41].

*(2) Alginate*. Alginate, or alginic acid, is an anionic linear polysaccharide found in algae and bacteria. It is commercially harvested from the cell walls of brown algae (Phaeophyceae), like *Laminaria hyperborea*, *Laminaria lessonia*, *Macrocystis pyrifera*, and *Ascophyllum nodosum* [27]. Alginate forms hydrogel by ionic cross-linking with divalent ions like calcium and zinc, thus ensuring the retention of cells and proteins within the hydrogel by 90% [42]. Similar to chitosan, purified alginates have a negligible immune response in vivo [43]. They are also biocompatible and nonthrombogenic [44]. Alginate-based hydrogels can also be modified to suit the host myocardium by molecular weight dispensation or by cross-linkage changes [43]. Alginate scaffolds alone had a substantial effect on the cardiac function of MI heart in rat, swine, and dog models, with no arrhythmia or thrombus formation. However, similarly to chitosan, the seeding of alginate with stem cells/fetal cardiac cells has been observed to have an enhanced effect on the ischemic heart of animal models [42, 45–47]. An investigation after 65 days post treatment with rat fetal cardiac cells (RFCC) in alginate scaffolds found that rat MI models had improved neovascularization, persistent fractional shortening, and end systolic and diastolic internal diameters and encouraged the formation of myofibers and cardiac gap junctions [48]. Correspondingly, when the RFCC was replaced by human embryonic stem cells (hESC) with an inhibited p38 mitogen-activated protein kinase, there was a significant improvement, and no immune response was noted [49]. Alginate is commonly modified by ECM-derived peptides like the arginine-glycine-aspartate (RGD peptide) sequence [42]. The RGD sequence is a signaling domain of fibronectin and laminin and thus assists the scaffolding in cell adhesion and signaling by binding ECM proteins to integrin receptors [43]. When compared with unmodified alginate, RGD-alginate showed a significant increase in the level of angiogenesis [50]. A comparative study, however, revealed that unmodified alginate shows a lower left ventricle expansion index, reduced left ventricle fractional shortening, and more scar thickness than RGD-alginate does [51]. The addition of a heparin-binding peptide to RGD-alginate, seeded with RFCC, stimulated a striated fiber organization similar to native tissue in vivo, but this was negative with RGD-alginate [52]. Aside from seeding alginate scaffold pores with cells, the use of a 3D nanocomposite of gold nanowires has also been indicated as it improves electrical communication between adjacent cardiac cells, leading to better cell organization, synchronous contractions, and higher levels of sarcomeric *α*-actinin and Cx-43 [53]. Other studies have shown that the use of a magnetic field of 5 Hz to stimulate alginate scaffolds filled with magnetically responsive nanoparticles (NPs) leads to increased troponin-T levels and a greater activation rate of AKT protein kinase [54]. Alginate was also identified as a structural complement of chitosan and combined for possible synergistic effect; the chitosan-alginate beads produced similar results with and without cell incorporation. It was also observed that alginate alone showed better results than did a chitosan-alginate combination [55]. Clinical trials involving alginate showed no significant improvement in ejection fraction and left ventricular end-systolic and end-diastolic volumes, though it did not deteriorate the status of the participants [56]. Some limitations have also been reported with the use of alginate as a biomaterial. Some authors describe cross-linked alginate to have poor long-term stability due to the ability of the gel to dissolve as a result of released divalent ions and exchange reactions. It is also speculated that the small pore size of alginate hydrogels (approximately 5 nm) could limit the number of regenerative mediators released [57].

*(3) Agarose*. Agarose is a repeating unit of D-galactose and 3,6-anhydro-L-galactopyranose, refined from algae. Agarose is well recognized as an enzyme stabilizer and a good culture medium for cells [58]. It possesses the ability to aggregate stem cells such as marine induced pluripotent stem cell (iPSC) and hESC due to its noncell-adhesive, transparent, and moldability characteristics [58]. The culture also showed potential for cardiac cell differentiation [58]. The application of agarose in cardiomyogenesis has not been fully elucidated upon as compared to other polysaccharides, though its role has been mentioned in the differentiation of stem cells to chondrocytes and dopaminergic neurons [59, 60].

*(4) Hyaluronic Acid*. Hyaluronic acid (HA), or hyaluronan, is a nonsulfated, high-molecular-weight GAG that is abundant in the ECM. It consists of repeating polymeric glucuronic acid and N-acetyl-glucosamine disaccharide conjugated by a glucuronidic *β* (1 → 3) bond and hexosaminidic *β* (1 → 4) bonds [61]. Without modification, HA has poor mechanical properties that limit its use as a biomaterial [62]. The most common modification of HA involves cross-linking, mediated by cross-linkers like cysteine derivatives, adipic dihydrazide, glutaraldehyde, carbodiimides, and divinylsulfone [63]. Additionally, comparative research indicated that the potential of HA in cardiomyogenesis depends on the molecular weight of HA and the evolution of MI [63]. The use of HA modified with polyethylene glycol-thiol injected into MI rat models showed a decrease in the size of the infarcted area and the rate of apoptosis, with a considerable increase in the number of arterioles and capillaries [64]. When combined with SF and seeded with rat MSCs, HA enhanced the expression of cardiac genes including Gata4, Nkx2.5, Tnnt2, and Actc1 in vitro. It was noted that the CD44 surface markers influenced the differentiation [65]. Combining HA and gelatin together with other chemical modifiers like activin-a, BMP-4, insulin, valproic acid, and 5-azacytidine in various combinations leads to the differentiation of human ADSC to CM with the expression of GATA4, TBX5, and cTnI [66].

#### 2.2.2. Protein-Derived Biomaterials in Cardiomyogenesis

They constitute as one of the major biomaterials used in cardiomyogenesis. These protein isolates retain their innate biological function, such as aiding differentiation, providing support, and assisting cell proliferation [9, 67]. They are one of the major scaffolds employed in cardiomyogenesis because of their role in cell relocation, multiplication, and differentiation of both CSC and other body stem cells to CM [68], as observed in studies showing the role of trade names Matrigel and Geltrex protein isolates in the reprogramming of mouse fibroblasts to CM [69]. Extracellular matrix protein isolates (ECMPi) were also observed to facilitate sarcomere alignment in human iPSC [70]. The remodeling of ECMPi, such as collagens into fibrils, has been linked with its effect in assisting the conversion of MSC to CM [71]. An in vivo investigation also showed that collagen and fibrin could increase cardiac function when implanted into the epicardium of rat models suffering from MI. Also, a percutaneous approach in larger animals has also been found to be supportive in MI [72].

*(1) Collagen*. Collagen is the most widely used natural polymer due to its ability to support an infarcted heart and improve cardiac function by inhibiting fibrosis, enhancing vascularization, and improving cell migration [73]. It is the most abundant ECM protein, and it provides structural scaffolding and tensile integrity and guides biological processes in tissue repair [43]. It contributes to the integrin interactions within the ECM, thus having a part in cell migration, cell differentiation, and tissue restoration [74]. It is classified as a nontoxic, nonimmunogenic, and biodegradable compound that is available commercially at a low cost [23]. Its viscosity also helps maintain MSC in the infarcted regions [72]. The native environment of CM has demonstrated collagen types I and III to be the main support for optimal cardiac functioning by providing structural sustenance and maintaining flexibility for contractile elements, respectively [75]. Nonfibrillar collagens, including collagen IV, V, and VI, have also been indicated in cardiac repair [76]. However, it has been reported that the collagen type I content in MI drastically reduced from 80% to 40% [77]. Studies prompted by this observation showed that acellular type I collagen in the form of a cardiac patch on MI murine hearts preserved contractility, prevented remodeling, and improved heart function, by reducing infarcted region fibrosis and supporting blood vessel formation [73]. Similar to other biomaterials, studies have described that the inclusion of other complexes to collagen could attenuate its potential [1]. However, studies done by Dawson et al. showed that the addition of the RGD peptide to type I/III collagen, seeded with mouse ESC-derived embryoid bodies, had no significant input in the potentials of collagen in CM formation [78]. The addition of carbon nanotubes to type I collagen hydrogels showed better results with increased cardiac cell functions compared to using pure collagen hydrogels. The authors suggested that the addition of carbon nanotubes to collagen hydrogels is recommended for future studies involving collagen to avoid mismatches in the mechanics, conductivity, and submicrometer structure of the matrix [79]. Similarly, Sun et al. attempted the use of collagen hydrogel combined with single-walled carbon nanotubes on neonatal rat ventricular myocytes and noticed a better cell alignment, stronger contraction potential, no CM toxicity, and enhanced cardiac constructs [80]. Carbon nanotubes aside, other studies have revealed that a collagen-gold nanocomposite (43.5 ppm)-coated catheter with MSCs enhances cellular migration and thus leads to improved neovascularization [81]. Likewise, vitronectin-collagen scaffolds have been shown to support neovasculogenesis and boost ventricular function [82]. The incorporation of chitosan with collagen has shown a decrease in tensile modulus (1.82 to 0.33 MPa) and an increase in compressive modulus (23.50 to 55.25 kPa). The action of collagenase was also reduced in the presence of chitosan [83]. When tested for its role in cardiomyogenesis, chitosan-collagen hydrogel incorporated with prosurvival angiopoietin-1-derived protein improved the left ventricular ejection fraction and left ventricular fractional shortening and decreased the systolic dimension and volume although it did not affect diastolic parameters. Also, the chitosan-collagen hydrogel did not slow the rate of apoptosis; as compared to nonmodified collagen hydrogel, however, it was able to enhance the survival of CM in the rat MI model [84, 85]. A comparative review on the role of cross-linking of collagen on cardiomyogenesis showed that noncross-linked scaffold obtained a higher biocompatibility and complete adhesion to the heart with a mild inflammatory reaction [43]. The role of collagen in cardiomyogenesis keeps on unfolding with the current attention it gets from researchers, and it remains one of the most studied biomaterials concerned with stem cell differentiation.

*(2) Fibrin*. Fibrin is a self-assembling peptide associated with clot formation in the endothelium. It functions as a biomaterial by having good seeding competence, efficient biocompatibility and biodegradability, uniform cellular migration, and efficient adhesion [86]. It is formed by the reaction of thrombin and fibrinogen. Thus, it could be harvested from the patient's blood, therefore minimizing immune rejection [74]. Fibrin can be manipulated to form hydrogels, microbeads, and gels [74]. It can also be manipulated to incorporate biological molecules like fibroblast growth factor and transforming growth factor-*β*1 to enhance the presence of growth factors and to resemble the native ECM of the heart [87, 88]. Its intrinsic regenerative properties give it the ability to be a stand-alone therapy [43]. A meta-analysis by other authors pointed out that fibrin glue, administered alone, reduced the infarct size and stimulated neovascularization more than when administered with neonatal skeletal myoblasts in rat MI models [43]. However, fibrin scaffolds incorporated with thymosin *β*4 (encapsulated in gelatin microspheres) significantly increased vascular growth and sustained the survival of swine MSC, thus producing a synergistic effect [89]. In vivo experiments showing the potential of human embryonic stem cell-derived cardiac progenitors (hESC-CPC) in nonhuman primate models when seeded on a fibrin patch led to the commencement of clinical trials accessing the possibilities of fibrin patch with hESC-CPC for individuals with heart failure. The clinical study is titled “Transplantation of Human Embryonic Stem Cell-Derived CD15+ Isl-1+ Progenitors in Severe Heart Failure” and can be located at https://clinicaltrials.gov/ct2/show/record/NCT02057900. It is expected to be completed in 2018 [90]. Fibrin gel has also been described to be an efficient sealant after intramyocardial injection, thus avoiding displacement of injected materials [91].

Advanced studies using fibrin hydrogel have also illustrated that synchronous electromechanical cell conditioning (2-millisecond pulses of 50 mV/cm at 1 Hz and 10% stretching for seven days) of cardiac ADSC before implanting into a murine heart might be an ideal strategy for future research [92]. Tao et al. also suggested seeding fibrin gels with 3 to 4 million neonatal cardiac cells from Sprague-Dawley rats to achieve optimal results for in vivo cardiac patch implantation. They speculated that seeding with quantities outside the given range may affect cell density on spontaneous contraction rates, contraction forces, and paced response frequencies [86]. Ichihara et al. recommended epicardial placement to intramyocardial injection when delivering fibrin glue incorporated with male bone marrow MSC, as this led to better initial retention and long-term presence of MSC. There was also the quicker recovery of cardiac function and structure as measured by echocardiography and catheterization in female MI rats [93]. Further studies have also shown that fibrin gel could be used in tissue engineering to form aortic valves with bioinspired textile reinforcement [94].

*(3) Gelatin*. Gelatin is a product of collagen hydrolysis. It involves the loss of the triple helical assemblies of collagen. It considered safe by the US Food and Drug Administration [95]. It is biocompatible and biodegradable and has a low antigenic level [43]. Gelatin hydrogels have flexible elastic moduli and can be cross-linked with transglutaminase to be thermostable [96]. Gelatin hydrogels have also been indicated to be preferred to alginate and fibronectin-patterned polydimethylsiloxane due to their performance in sustaining neonatal rat cardiac myocyte tissue in vitro for three weeks at maintained spontaneous beating, higher spare respiratory capacity, and consistent levels of contractile stresses [97]. Gelatin hydrogel-muscular thin films also supported the growth of human iPSC-derived cardiac myocytes [97]. Results of the Autologous Human Cardiac-Derived Stem Cell to Treat Ischemic Cardiomyopathy (ALCADIA) experimentation illustrated that 200 *μ*g of bFGF in a biodegradable gelatin hydrogel sheet implanted on the epicardium of human patients with ischemic cardiomyopathy and heart failure leads to the continuous release of bFGF, thus suggesting the effectiveness of the methodology [98, 99]. Ultraviolet cross-linkable gold nanorod-incorporated gelatin ethacrylate hybrid hydrogels seeded with neonatal rat ventricular CM exhibited excellent cell retention, viability, and metabolic activity [100]. There was also a synchronous beating of the CM [100]. Comparative studies involving a pig MI model also demonstrated that gelatin scaffold + bFGF + human cardiosphere-derived cells had a higher ejection fraction, lower infarct volume, and more cellular differentiation to CM, than when compared to the use of gelatin scaffold + bFGF and gelatin scaffold + bFGF + human bone marrow-derived MSCs [98]. The use of gelatin as a scaffold for cardiomyogenesis has been adopted as a standard protocol for further investigations. For example, Kudová et al. demonstrated that mouse ESC on gelatin-coated dishes had poor CM differentiation when hypoxia-inducible factor-1*α* was deficient in the medium in vitro [101]. Bioartificial constructs constituting polylactic-co-glycolic acid and gelatin to resemble the anisotropic structure and mechanical characteristics of the myocardium indicated that the combination leads to good involvement of human MSCs to form CM due to the presence of markers, namely, Gata4 and Mef2c [102].

*(4) Matrigel*. Matrigel is a protein isolate from the basement membrane of Engelbreth-Holm-Swarm mouse sarcoma cells [103]. It is predominantly composed of laminin with other components including heparin sulfate proteoglycan, entactin, and collagen type IV. Studies revealing Matrigel's robustness in containing growth factors such as bFGF, epidermal growth factor (EGF), IGF-1, PDGF, nerve growth factor (NGF), TGF*β*1, and others have prompted researchers to investigate its potential in cardiomyogenesis. However, information on its mechanical and chemical behavior has not been fully reported [38, 43, 104]. Comparative and descriptive reviews have already illustrated the role of Matrigel in the treatment of MI by enhancing the recruitment of CD34^+^ and c-kit^+^ stem cells in mouse models, enhancing CM function in vitro, and serving as an efficient scaffold for pluripotent stem cells and embryonic stem cell delivery and integration [9, 37, 43, 74]. Current studies using Matrigel have included an advanced investigation. Zhang et al. demonstrated that seeding Matrigel with CSC from embryonic heart tubes could differentiate into cardiac peacemaking cells after endothelin-1 treatment, thus forming a tissue-engineered cardiac pacemaker. When combined with endothelial stem cells and introduced in vivo in rat models, there was enhanced vascularization and electrical activity [105]. Matrigel also enhanced the type I collagen matrix due to its type IV collagen and sulfated proteoglycan content, thus enhancing the mechanical culture of heart valve interstitial cells in vitro [106]. However, comparative studies show that graphene promoted the differentiation of hESC to CM than Matrigel did in vitro [107]. The comparative studies were prompted by different opinions on the use of Matrigel or a “mouse tumor” derivative in human infarcted tissue [108]. Since there is also poor control on the cells that secrete the Matrigel components, matrigels from various Engelbreth-Holm-Swarm mouse sarcoma cells may also lead to matrigels with varying qualitative and quantitative differences, thus altering their role in cardiomyogenesis.

*(5) Cardiogel*. Cardiogel is an ECM matrix, synthesized by cardiac fibroblasts. It contains laminin, fibronectin, types I and III collagen, growth factors, and proteoglycans [109]. The synergistic effect of these components has been reported to influence the growth of CM, enhance spontaneous contractile activity, and stimulate stem cell differentiation [109, 110]. The debate concerning whether the components of cardiogel support cardiomyogenesis, more than the complex cardiogel itself, led to comparative studies which suggested that simple matrices will be ideal for structural and biochemical investigation; however, native complex matrices like cardiogel overwhelm cell polarity faster than do simple mediums and will also produce favorable results [111]. Similarly, culturing MSCs from rat models on a 3D matrix, cardiogel was better in cellular expansion and adhesion than were MSCs cultured on plastic and fibronectin-coated plates [112]. Previous reviews also pointed out that cardiogel protects murine bone marrow-derived mesenchymal stem cells (BMSC) from oxidative stress better than does Matrigel [113]. Another study also reported BMSC differentiating to cardiogenic components without induction with 5-aza, thus emphasizing the potential of cardiogel. However, the authors could not provide a good explanation for their result [114]. Cardiogel seeded with ASCs has also been shown to support angiogenesis in vivo [115]. A proper explanation of the biochemical and mechanical role of cardiogel is still needed for proper direction of how cardiogel could be manipulated for optimal results.

*(6) Decellularized Extracellular Matrix*. Decellularization involves the removal of cells and nuclear materials from organic tissues while making possible efforts at not tampering with the structural integrity or native components of the ECM [74]. Similarly to ECMPi, they are biodegradable and safe byproducts. The major reason to decellularize is to minimize immune reaction when used [9]. The idea to use decellularized matrices (DECM) was also due to the lack of a native architectural framework in other biomaterials [24]. DECM could be in the form of an intact whole organ, small tissue sections, thin sheets, hydrogels, or coating [116]. Although the potential for the use of DECM in cardiomyogenesis is undisputable, several authors have initiated discussions concerning its uses. Kim et al. described that DECMs might possess remnant molecules ranging from microRNA to cellular proteins, and thus, they suggested that DECMs should have less than 50 ng of dsDNA per mg dry weight and less than a 200-base pair DNA fragment length before use [117]. Decullarization methods have also been reported to affect the architecture of tissues. For example, decellularization by freeze-thawing could affect the ultrastructure of the ECM; pressure techniques could affect their mechanical properties; solutions with high or low pH could degrade some ECM components; alcohol could cross-link collagen, making it stiff; and ionic detergents could also disrupt covalent bonds [118–122]. Thus, some reviews have suggested that the type of tissue (such as heart, kidney, or liver) and the need for decellularization should be considered before selecting a decellularization protocol [116]. Another study reported that the current decellularizing protocols have also led to residual fragments of Triton X-100, sodium deoxycholate, and sodium dodecyl sulfate (SDS) in decellularized scaffolds, thus affecting their functionality [123]. This illustrates that proper clearance of the reagents used in protocols is necessary for optimal results. Several protocols for the decellularization of the human myocardium have also been tested, and suitable protocols have been derived [124]. Other studies have shown that CPCs isolated as cardiosphere-derived cells thrive better on a healthy DECM than on a pathologic DECM [125]. Opinions on decellularization can be found in very detailed reviews [116, 117, 123, 124, 126–128].

While the subject of decellularization has been elucidated upon, studies on the role of DECM in cardiomyogenesis have been going on concurrently. Over the past few decades, several studies have been conducted and mentioned in reviews [9, 24, 43, 74]. Recent studies include descriptions of how DECM sheets produced from thin, cardiac sections from rat neonatal ventricles efficiently preserved and improved phenotypic characteristics and cell proliferation and viability rates of CM in vitro [129]. MSC and iPSC cultured on DECM from the left ventricle of humans were shown to have good adhesion, proliferation, and viability compared to cells cultured in a purchased cardiac myocyte medium [130]. Repopulating decellularized mouse hearts with human iPSC-derived multipotential cardiovascular progenitor cells showed signs of CM differentiation, proliferation, and myofilament formation [131]. Nonseeded decellularized homografts, derived from donated human heart valves, were also able to reduce complications associated with bovine jugular vein conduits and cryopreserved conventional homografts [132]. The addition of fibronectin to enhance the adhesion of human cardiovascular cells to a decellularized porcine heart scaffold for proper proliferation and integration was also reported [133]. Recellularization of decellularized rat heart with isolated rat CM showed the expression of CD31, *α*-actinin, troponin-T, connexin, Nkx-2.5, c-kit, and GATA4. The electric potential was detected on the scaffolds, and the cardiac pacing was also observed on the scaffold [134]. Further studies also showed that surface heparin treatment tends to reduce tissue calcification of the decellularized porcine heart valve in a rabbit intramuscular implantation model [135]. Hodgson et al. also developed a protocol to ensure 98% decellularization of the whole porcine heart with reduced time of exposure to detergent [136].

### 2.3. Synthetic Biomaterials

The use of synthetic biomaterials in cardiomyogenesis was prompted because of the need to develop polymers that are easy to fabricate and manipulate, thus enabling the production of biomaterials for specific stem cell response [9]. The possibility of having polymers with a steady manufacturing process and the opportunity to construct their physical properties also attracted researchers to the potential of synthetic materials. The ability to also influence their molecular weight, heterogeneity index, and copolymerization ratio to control their degradation speed has also caused researchers to develop greater interest [74]. Since the involvement of synthetic biomaterials in cardiomyogenesis, their prospects have been climbing. However, their use is limited by poor bioactivity, potential toxicity, and low interaction with cells and signaling proteins. Thus, their ability to sustain cells has not reached the levels attained by natural biomaterials [137]. This setback has led to combining synthetic biomaterials with natural biomaterials so that the resulting scaffold interacts properly with cells [138].

Some of the synthetic biomaterials used in cardiomyogenesis include caprolactone and derivatives, polyglycolic and polylactic acids and derivatives, polyurethane, self-assembling peptides, carbon nanotubes, and polyketals [24].

Caprolactone is considered nontoxic and tissue compatible with good pH sensitivity. However, it is difficult to synthesize and degrades slowly. Polyglycolic and polylactic acids and derivatives have good biocompatibility and degrade easily, although they acidify their environment when degrading, which could lead to erosion. Polyurethane is biocompatible but nonbiodegradable unless copolymerized; it also lacks conductivity. Self-assembling peptides are bioreabsorbable and can be used to design 3D microenvironments. Their toxicity profile and side effects still require more elucidation. Carbon nanotubes provide good conductivity and mechanical support, but they are hydrophobic, toxic, and expensive. Polyketals are cheaper, nonimmunogenic, inert degradation products and are sensitive to low pH. Their use is limited by complexity in the synthesis and quick macrophage uptake and degradation [139–144].

Poly(*ε*-caprolactone) combined with poly(L-lactic acid) and collagen to form a nanostructured matrix supported the isolated rabbit CM with results similar to those expected in the native myocardium [145]. Conductive polymers like polypyrrole have been combined with poly(*ε*-caprolactone) and gelatin to form nanofibrous membranes, and these membranes supported human CM attachment, proliferation, and interaction [146]. The biodegradable patch composed of poly(l-lactic-co-*ε*-caprolactone) and polyglycolic acid also supported human-induced pluripotent stem cell-derived CM in regenerating a host myocardium in the athymic rat [147]. The ultrafine fiber scaffold made by combining the additive manufacturing of poly(hydroxymethyl glycolide-co-*ε*-caprolactone) with melt electrospinning writing showed that it aligned the growth of cardiac progenitor cells in the direction of the melt electrospun and had better results than using electrospun poly(*ε*-caprolactone)-based scaffolds alone [148]. The rapamycin-loaded polylactic-polyglycolic acid NPs delivered locally in minipigs considerably reduced the MMP-2/TIMP-2 ratio and proliferating cell nuclear antigen expression, increased the p27 (kip1) mRNA expression, thus relieving the degree of stenosis, and showed excellent acute procedural results in the interventional coronary artery-oversized balloon injury model [149]. The electroactive polyurethane/siloxane films containing aniline tetramer moieties (EPUSF) supported the proliferation and differentiation of C2C12 myoblasts with the expression of cardiac-specific genes of HL-1 cells involved in muscle contraction and electrical couplings such as Cx43, TrpT-2, and SERCA genes. The EPUSF were nontoxic and did not alter the intrinsic electrical characteristics of HL-1 cells [150]. 3D biomimetic scaffolds using a polymer blend of polyurethane and cellulose were also reported to have good biocompatibility, provided good mechanical support, and housed frequent contraction cycles of cardiac tissue [151]. Soft polyurethane-urea scaffolds with regular tubular pores were also observed to withstand tensile stresses associated with diastole without opposing tissue contraction. Also, they supported the growth of cardiac myocytes than did tissue culture plastic. Enhanced seeding efficiency was further noted [152]. A comparative study also showed that poly(L-lactic acid) and polyurethane nanofibrous mats fabricated by solution blow spinning were better substrates for cardiac cell culture than polystyrene was [153]. The role of carbon nanotubes has been described in this review [32, 79, 81]. Other studies have gone on to demonstrate that embedding carbon nanotubes in mouse embryoid bodies to control mechanical and electrical activity in stem cell niches led to cardiac differentiation and beating activity [154]. Polyketals have been employed due to their efficient role as a vehicle for the delivery of molecules. Studies on polyketals showed that they serve as good vehicles for delivering siRNA to an MI heart, thus posing as a technique in targeting oxidative stress [155].

It will be interesting to note that synthetic biomaterials are currently being well utilized in the field of nanotechnology. The use of nanotechnology in cardiomyogenesis is reviewed in the next section. The current natural and synthetic biomaterials in cardiac regeneration field which are discussed in this review are summarized in the Table 1. Furthermore, they are classified based on the type of reported experimental studies.

### 2.4. Nanotechnology in Cardiomyogenesis

The use of nanotechnology has advanced significantly in the medical field in recent years. Nanotechnology can generally be described as the manipulation of matter on an atomic, molecular, or supramolecular scale, and it can also be described as the understanding and manipulation of processes in structures having sizes ranging between 1 and 100 nanometers (nm) [156, 157].

**Figure 2:**
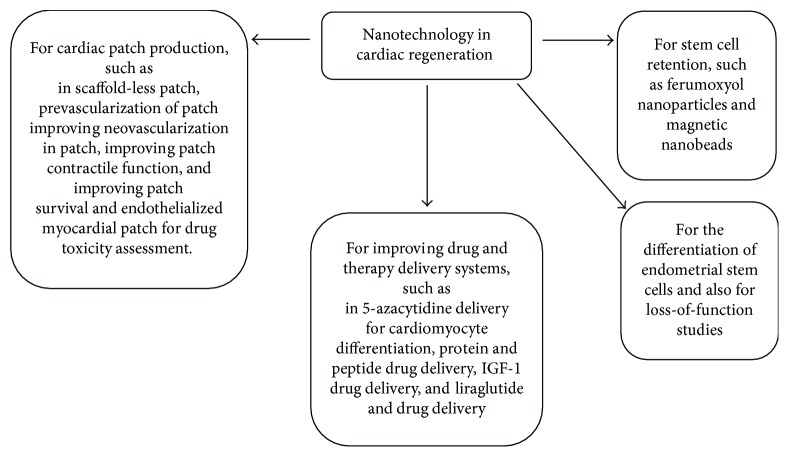
Application of nanotechnology in cardiomyogenesis.

NPs can be produced by using different composite materials, thus altering their physical properties. Some of the materials used in the production of NPs include gold, silver, iron, titanium dioxide, cerium, silica, carbon, copper, zinc, nickel, and magnesium, among others [156]. Among all these materials, gold, because of its hydrophobic nature, has been shown to be most favorable in the construction of NPs. De la Fuente et al., in 2001, reported the first water-soluble AuNPs [158]. Various factors are considered in application of NPs, such as their physical properties, biocompatibility, and cytotoxic effects [156]. Based on these properties, NPs have various applications in the cardiomyogenesis filed which are summarized in the Figure 2.

Nanotechnology incorporates diverse novel, powerful tissue engineering techniques such as 3D bioprinting, and studying these techniques has shown great prospects [159]. In 3D bioprinting, biomaterials, cells, drugs, growth factors, and genes are deployed in a layered manner to produce a 3D construct regarded as a “bioink” [160–163]. Technology has also evolved to enable the creation of scaffold-free or scaffold-based tissues and organ constructs [164–168].

Three main modalities are used in bioprinting: laser, droplet, and extrusion-based bioprinting, of which droplet-based bioprinting (DBB) provides several advantages because of its simplicity, versatility, and agility and the enormous level of control over the pattern of deposition [169]. Current DBB methods include inkjet, acoustic, electrohydrodynamic, and microvalve bioprinting [169]. Regardless of its vast benefits, this technology still faces challenges, such as bioprinting-triggered cell damage at significant levels, limited bioink materials, bioprinted constructs with limited structural and mechanical integrity, and size restrictions of constructs due to lack of porosity and vascularization [169].

3D bioprinting in the cardiovascular field has been reviewed with respect to biomaterial dependence or independence. Moldovan et al. concluded that biomaterial or scaffold-dependent bioprinting was appropriate for tasks needing faster, larger, anatomically correct, matrix-rich, and cell-homogenous constructs while scaffold-free bioprinting is suited for complex and smaller constructs with poor matrices, longer time preparation, and cell-heterogeneous components [159]. Currently, the interface of scaffold-free and scaffold-dependent bioprinting is the utilization of a new generation of bioinks exclusively prepared from natural materials, like collagen, fibrin, and other organ-specific extracellular matrices [170].

The diverse potentials and applications of nanotechnology in cardioregeneration have been shown by several studies which shall be briefly considered in this review.

#### 2.4.1. Cardiac Patch Production

*(1) Scaffold-Less Patch*. Native cardiac tissue simulation requires that the CMs are closely packed with adequate electrical connections between cells maintained by the nexus (gap junctions). However, cardiac tissue construction with scaffolds attenuates these cell-to-cell-nexus interactions, and also the scaffold biodegradation can also result in inflammation [171]. Several methods have been considered for the production of scaffold-free cardiac tissue, and this includes grafting poly(N-isopropylacrylamide) to a culture surface that is thermoresponsive [172]. Another is the spheroids, which is a scaffold-free, tissue-like aggregation of cells [173].

Shimizu et al. in 2007 developed a technology which they termed “Magnetic force-based tissue engineering (Mag-TE),” which was used to construct contiguous sheets of CM [171]. Original magnetite cationic liposomes (MCLs) with a magnetite NP (Fe_3_O_4_) content of 10 nm were used in the study. The CM construct was shown to be a magnetically aligned functional cluster, the presence of the gap junction protein connexin 43 was demonstrated, and no toxicity was demonstrated in the construct after 24 hours of incubation [171]. However, the study did not consider the required uptake of MCLs, as well as the impact of the magnetic force.

*(2) Prevascularization of Patch*. Although the cell patch platform has shown successful results in cardiomyogenesis, key barriers such as low biophysical integration and a lack of organized vascular plexus still need to be overcome to achieve the high level of functional repair needed for treating myocardial injury [174]. Having this background challenge in mind, Jang et al. designed a “3D printed prevascularized stem cell patch,” which they proposed would enhance tissue regeneration and repair by promoting speedy vascularization post patch transplantation [175]. The study utilized dual stem cells (CSC and MSC) spatially patterned on a decellularized extracellular matrix. The printed structure was shown to improve cell-to-cell interactions and differentiation capabilities. The patch also enhanced cardiac functions by reducing cardiac hypertrophy and fibrosis. Migration from the patch to the area of infarct was enhanced, and cardiomyogenesis and neovascularization were demonstrated at the injured myocardium [175]. However, the study did not look into the effects of structural parameters like the line width of the construct, the number of required cells and ratio of cells, and the in vitro conditioning of the prevascularized stem cell patch.

*(3) Improving Neovascularization in Patch*. As previously mentioned, neovascularization and organized vascular networks remain a challenge to the clinical application of the cardiac patch. The impaired nutrient supply and oxygenation perfusion post myocardial infarction will cause the regenerated cardiac tissue to be restricted to a particular zone with only a marginal improvement in function. Thus, Gaebel and colleagues designed a cardiac patch by using the laser-induced forward transfer (LIFT) cell printing method [176].

In the study, they prepared a polyester urethane urea (PEUU) cardiac patch which was seeded with hMSC and human umbilical vein endothelial cells (HUVEC) in a defined pattern. The LIFT-fabricated and controlled patches (where an equal quantity of cells was randomly seeded without LIFT) were transplanted into an infarcted rat heart, and cardiac performance was measured eight weeks post infarction. The study demonstrated that the LIFT-derived patches increased vessel formation, enhanced the capillary density, and thus significantly improved the function of the infarcted hearts [176].

*(4) Improving Patch Contractile Function*. We also considered the expected weakness in the contraction force of myocardial cells in the region of the heart that has been injured. As such, Fleischer et al. sought for ways to improve the performance of the cardiac patches that were transplanted for therapy [177]. It has accordingly been shown that there is a unique subpopulation of coiled perimysial fibers within the natural heart matrix [178]. These fibers have been said to provide the heart with the unique mechanical properties needed for efficient and continuous contractions as these fibers stretch and recoil with the CM [178, 179].

Fleischer et al created a nanocomposite coiled fiber scaffold, which was incorporated into the coiled fiber scaffold for heart tissue engineering [177]. The study utilized poly(ε-caprolactone), dichloromethane, and dimethylformamide which were electrospun to fabricate the coiled fibers, after which gold was deposited on their surface to create the nanocomposite. The electrospun fiber structure resembled the previously described perimysial fibers [177].

The study demonstrated that the addition of AuNPs to the scaffolds caused a rapid fabrication of elongated and aligned heart tissues with a similar morphology to that of cardiac cell bundles in vivo [177]. The study further demonstrated that the coiled fiber scaffolds demonstrated a stronger force of contraction, increased beating rates, and reduced excitation thresholds when compared with tissues grown within straight fiber scaffolds [177]. Hence, the conclusion is that this construct can be used to engineer cardiac tissues with superior functionality within diverse types of biomaterial scaffolds [177].

Likewise, in the bid to create a more functional cardiac patch that is capable of stronger contractile functions, Ravichandran et al. proposed a “gold nanoparticle-loaded hybrid nanofiber” for myocardium tissue regeneration [180]. The study sought to create a hybrid scaffold that can couple mechanical, electrical, and biological properties desired for cardiomyogenesis [180]. In the fabrication of the scaffold, the study used BSA/PVA scaffolds embedded with AuNPs by electrospinning (BSA is a water-soluble transporter protein of important physiological ligands, while PVA is polyvinyl alcohol, which is a water-soluble synthetic polymer). The study showed that differentiated cells on AuNP-loaded nanofibers expressed the cardiac proteins troponin-T, actinin, and connexin 43 and also exhibited the characteristic multinucleated morphology.

*(5) Improving Patch Survival*. Gaetani et al., while considering poor cell engraftment and significant cell death post transplantation, designed a cell patch targeted at increasing cell retention and survival [181]. The study looked into the therapeutic possibilities of a 3D-printed cardiac patch fabricated from human cardiac-derived progenitor cells in a matrix base of HA/gelatin. The 3D-printed biocomplex was transplanted into a myocardial infarcted mouse model, leading to cardiac performance preservation and remarkable reduction in adverse cardiac remodeling [181]. Over the 4-week follow-up period, the matrix also showed a prolonged in vivo cell survival and engraftment with a demonstrable temporal increase in heart and vascular differentiation markers.

*(6) Endothelialized Myocardial Patch for Drug Toxicity Assessment*. Zhang et al. also proposed a 3D bioprinting-based novel hybrid strategy in the fabrication of an endothelialized myocardium [182]. In the study, endothelial cells were directly printed within hydrogel scaffolds, and the 3D endothelial bed was thereafter seeded with cardiac cells to create an aligned myocardium, capable of spontaneous and synchronous contractions [182]. The CM greatly expressed proteins obligatory for proper cardiac contractile function. Also, the CM expressed well-aligned sarcomeric banding with a large number of gap junctions, thus providing the histologic basis for the synchronous contraction of the cardiac construct [182].

They further went on to create an “endothelialized-myocardium-on-chip platform” by embedding the construct into a specially designed bioreactor; this was to assess for cardiovascular toxicity. The chip construct was exposed to doxorubicin, a common anticancer drug. This resulted in the reduction in the beating rate of the cardiac cells, while the control maintained a relatively high beating rate. Likewise, there was a reduction in the levels of the Von Willebrand factor secreted by the endothelial cells relative to the control [182].

#### 2.4.2. Improving Drug and Therapy Delivery Systems

*(1) 5-Azacytidine Delivery for Cardiomyocyte Differentiation*. A variety of nanomaterials have been employed as nanocarriers and have been used successfully in drug delivery, and this includes gold nanorods [183], graphene NPs [184], quantum dots (GQs) [185], mesoporous silica nanoparticles (MSNs) [186], and graphene NPs [184]. The safest of the investigated nanomaterials are the MSNs because of their low toxicity, tunable particle size and pore diameter, high loading potentials, incomparable biocompatibility, and multifunctional surface properties [187, 188].

Cheng et al. utilized the MSNs to deliver the drug 5-azacytidine to regulate the differentiation of P19 cells into CM. P19 cells are teratocarcinoma-derived and have been extensively used to model cardiac cell development and CM differentiation for cardiac repair [189]. Drugs like dimethyl sulfoxide (DMSO), retinoic acid, butyrate, and 5-azacytidine can cause P19 cells to differentiate into CM; however, a major limitation to the use of P19 cells is its low efficiency of differentiation [189].

They further went on to investigate if there would be any significant difference in P19 cell differentiation into CM if 5-azacytidine were delivered differently. In the study, 5-azacytidine was delivered using fluorescein isothiocyanate isomer I mesoporous nanoparticles (FMNs) and poly(allylamine hydrochloride) (PAH); the construct was coined FMNs + 5-azacytidine + PAH nanocomplex [189]. It was reported that 5-azacytidine delivered by FMNSs demonstrated a high induction efficiency than did 5-azacytidine alone [189].

*(2) Protein and Peptide Drug Delivery*. The growing attention and demand on various protein and peptide drugs for treatment purposes is serving as a driver for an increasing need for efficient delivery carriers of these drugs. Currently, polymeric NPs are being considered the most preferred and suitable means of sustained delivery of protein and peptide drugs [190]. Studies have shown that polyesters such as poly(lactide-co-glycolide) possess some intrinsic shortcomings as they are said to be more hydrophobic than the majority of the protein drugs deemed for encapsulation; also, a lot of stability problems have been associated with the protein drugs during storage and release [191]. Also, various liposomal formulations have been developed for the delivery of protein drugs and considered for clinical applications [192]. However, the application of liposomes clinically is not without disadvantages, including instability [193] and a short half-life due to rapid uptake by the reticuloendothelial system [194, 195]. A number of studies subsequently looked into the characterization of “polymer-supported liposomal systems,” with attention given to triblock copolymers (pluronic), which are copolymers of poly(ethylene oxide)-poly (propylene oxide)-poly(ethylene) (PEO-PPO-PEO) [196, 197]. Subsequently, a protein delivery system combining pluronic-based micelle and liposomal systems was developed and designed as a core/shell NP with a lecithin core loaded with a growth factor and a pluronic shell [198, 199].

Oh et al. reported the fabrication of a temperature-induced gel made up of core/shell NPs [190]. The construct was made of a core of lecithin loaded with a vascular endothelial growth factor (VEGF) and a shell made of a pluronic F-127 (PEO-PPO-PEO) triblock copolymer. The addition of capryol 90 (propylene glycol monocaprylate) to the core/shell NP aqueous solution resulted in the formation of a temperature-induced gel of core/shell NPs at body temperature [190]. This construct was produced to enhance stable localization and sustained release of therapeutics by core/shell NPs at the site of the ischemia. The construct was injected into a myocardial infarcted rat model, and a functional analysis of the heart was performed after four weeks. The result showed that the VEGF-loaded core/shell NP with a gel construct improved cardiac functions, especially concerning cardiac output and ejection fraction.

*(3) IGF-1 Drug Delivery*. Insulin-like growth factor-1 (IGF-1) has been shown to induce Akt phosphorylation in cultured CM, thus preventing apoptosis of CM [200]. Although it has been proven that IGF-1 can be used in the treatment of both acute and chronic MI, it has equally been reported that prolonged overexpression of IGF-1 results in a reduction of the functional recovery of the heart [201], hence the need for a mechanism that can accurately control the release of IGF-1. This led to the study by Chang et al., in which they complexed IGF-1 with poly(D,L-lactide-co-glycolide) (PLGA) NPs (PLGA-IGF-1 NPs) [200]. The study reported that the PLGA-IGF-1 NP complex showed increased IGF-1 retention, induced the phosphorylation of Akt, and provided early cardioprotection post myocardial infarction when compared to IGF-1 alone [200].

*(4) Liraglutide Drug Delivery*. A recent study by Qi et al. evaluated the long-term retention and therapeutic effects of Liraglutide, a drug developed for type 2 diabetes treatment, on cardiac regeneration [202]. In the study, liraglutide was loaded in poly(lactic-co-glycolic acid)-poly(ethylene glycol) (PLGA-PEG), and this resulted in an efficiently loading and sustained release of bioactive liraglutide. The reported therapeutic effects of liraglutide on the heart include improved cardiac performance [202] and enhanced myocardial blood flow [203], inhibition of CM apoptosis [204], attenuation of infarct size [205], and myocardial signaling pathway activation [206]. The cardioprotective effect of liraglutide has been ascribed to several factors. These include the promotion of glucose metabolism over the metabolism of fatty acid which results in decreased oxygen demand [207], increased vascularization [208], and reduction of myocardial apoptosis [205]. However, the short half-life (13 hours) of liraglutide limits its clinical applicability, thus necessitating repeated subcutaneous injections [209, 210].

The biomaterial PLGA has been demonstrated to be a potential vehicular candidate for maintaining the local concentrations of proteins by sustained release for the treatment of cardiac diseases [211, 212]. Meanwhile, PEG prevents the phagocytosis of NPs by allowing them to evade the immune system [213, 214]. From the study, liraglutide thus benefitted from the advantages offered by the combined PLGA-PEG delivery system.

In all, Qi et al. demonstrated that the intramyocardial injection of NP-liraglutide in a rat model of myocardial infarction sufficiently improved cardiac function, attenuated the infarct size, preserved myocardial wall thickness, prevented myocardial apoptosis, and promoted angiogenesis when compared with the control that had an intramyocardial injection of saline [212]. Another positive side is that the glucose levels were not altered in the rat model [212].

#### 2.4.3. Nanoparticles and Magnets: Role in Stem Cell Retention

*(1) Ferumoxytol Nanoparticles*. A major limitation to the therapeutic effect of stem cell transplantation is the prevalence of low retention and engraftment rates [113]. Some studies have sought for ways to enhance cell retention and engraftment using NPs. One of those few studies includes the work done by Vandergriff et al. where ferumoxytol NPs in the presence of heparin and protamine was used to label human cardiosphere-derived stem cells [215]. The cardiosphere-derived stem cells labeled FHP were infused into syngeneic rats through the coronary vessel with magnetic targeting and without magnetic targeting as the control. This technique of augmenting acute cell retention by magnetic targeting resulted in attenuation of left ventricular remodeling and better therapeutic benefit such as improved ejection fraction three weeks after therapy. Histological sections showed enhanced engraftment of cells and angiogenesis in the cardiac tissues of the magnet-targeted group [215]. The study also demonstrated that FHP-magnetic targeting did not cause any iron overload or exacerbate cardiac inflammation and hence was said to be safe to cardiac stem cells [215]. However, further studies need to be done to determine the time window when stem cells can be reliably tracked using ferumoxytol labeling [215].

*(2) Magnetic Nanobeads*. A previous study using magnetic NPs is the work done by Zhang et al. where the human VEGF (hVEGF) gene was encoded in adenoviral vector (Ad)/magnetic nanobeads (MNBs), and the control of an external magnetic field was used to investigate its regenerative function on the hearts of rat models with acute myocardial infarction [216]. The complex, termed MNB/AdhVEGF, was injected intravenously with a magnet applied epicardially serving as an attractant for the circulating magnetic nanobead complex. The MNB/AdhVEGF complex when compared with the control resulted in a 50-fold higher therapeutic gene expression in the ischemic area of the heart. Also, over the control group, the MNBs/AdhVEGF complex group showed significant improvement in left ventricular function and also demonstrated higher arteriolar and capillary density with reduced collagen deposition [216].

### 2.5. Differentiation of Endometrial Stem Cells

Two important challenges with myocardial tissue engineering include the selection of a suitable cell source and the induction of angiogenesis [217]. Bioactive glass has been reported to affect angiogenesis, but the knowledge of its effect on soft tissue is not sufficient [218, 219]. The human endometrial stromal cells have been put forward as a rich and readily available resource in regenerative medicine. Barabadi and colleagues investigated the capacity of the endometrial stem cells to differentiate into the CM lineage in vitro. The study also evaluated the capability of bioactive glass NPs on hydrogel scaffolds to induce the differentiation of endometrial stromal cells into the endothelial lineage and to induce angiogenesis [217]. The report suggested that endometrial stem cells can be conveniently programmed into CM and are a suitable candidate for myocardial regeneration. Also, the study demonstrated improved angiogenesis [217].

### 2.6. Loss-of-Function Studies

The zebrafish has become an important model for studying myocardial regeneration because of its remarkable regenerative capacity. However, for the adult heart, loss-of-function studies are limited by effective gene knockdown and conditional knockout techniques [220]. Kikuchi et al. demonstrated the activation of Aldh1a2 in the endocardium and epicardium and showed that retinoic acid signaling is crucial for CM proliferation during zebrafish cardiac regeneration [221]. Also actively involved in the myocardial regeneration in zebrafish are the Gata4-GFP myocytes [221]. Diao et al. reported a novel siRNA knockdown technique using NPs of poly(ethylene glycol)-b-poly(D,L-lactide) (PEG-PLA) [220]. The siRNA-encapsulated NPs were delivered intrapleurally, and afterward, they transferred the siRNA into the cardiac tissue while avoiding the endosomes. This resulted in significant gene-specific knockdown effects in the adult heart exhibited by downregulation of the Aldh1a2 and Dusp6 proteins [220]. This downregulation sufficiently inhibited myocardial proliferation and reduced the number of Gata4-positive cardiac cells when compared with the control, suggesting that retinoic acid signaling was compromised by siAldh1a2 therapy [220].

### 2.7. Tissue Engineering in Cardiovascular Regeneration

The introduction of tissue engineering in cardiovascular regeneration has led to better understanding of the cardiac ECM and its constituting cells like cardiomyocytes and fibroblasts. It has also illustrated the problem with the association of functional active biomaterials with mathematical models [222]. Engineered 3D cardiac tissue constructs (ECTCs) possess the ability to imitate a complex cardiac physiology under optimal and pathological conditions [223]. ECTCs have also been gaining more attention than traditional 2D cell cultures have by being more cost-effective, by supporting vessel formation, and by accurately duplicating in vivo cell and tissue functions of cardiovascular structures [224]. Despite its promising role in cardiac regeneration, there are few ECTCs translated to the clinic, and the major reason has been linked to inconsistency in result performed in clinical trials [225]. Another setback is the difficulty in providing a metabolically appropriate environment in ECTCs since diffusion alone cannot sustain healthy cells. The current introduction of bioreactors to create a good environment for ECTCs has also improved some of the setbacks associated with ECTCs. However, these bioreactors are complex to use, unreliable, not cost-effective, and limited in functions [226]. Currently, studies are still done on ECTCs to ensure a stable and efficient transition from bench to humans that are stable for use. Some of these studies have identified the potentials of the “I-wire” platform in controlling the applied force on ECTCs while cross-examining their inactive and active mechanical and electrical characteristics, which can be vital in ECTC production [223]. The I-wire platform in ECTCs has also proved vital in examining cardiomyocyte mechanics during auxotonic contraction [222]. Other current reports have also shown new modifiable production processes for rapid fabrication of fibrous, semilunar heart valve scaffolds by varied parameters for biomimetic heart valve replacement which lasted for 15 hours in an ovine model [227]. The current application of nanotechnology and tissue engineering in cardiac regeneration which are discussed in this review are summarized in the Table 2. In addition, they are classified based on the type of reported experimental studies.

## 3. Pulmonary System and Diseases

The respiratory system is a highly complex operation which is comprised of the airways (categorized into the conduction sector and the respiratory sector) and the respiratory muscles such as the diaphragm. The conducting portion transports and humidifies the air and includes the trachea, bronchi, and bronchioles up to the terminal bronchioles. At the same time, the respiratory portion is involved in the actual exchange of gas, which includes the respiratory bronchioles, alveolar ducts, and the alveolar sacs.

There are over 40 pathological conditions identifiable with the airways [228]. However, of primary concern are end-stage pulmonary problems such as lung cancers, cystic fibrosis, pulmonary hypertension, and fibrosis and the increase in the use of tobacco. Especially in developing countries, this behavior is resulting in a surge of chronic, obstructive pulmonary diseases (COPD) [229]. Treatment options that are available for acute end-stage pulmonary failures are currently limited to mechanical ventilation and extracorporeal membrane oxygenation (ECMO) [229]. However, these treatments can only temporarily sustain the patient until it is possible to perform a lung transplant. We know, unfortunately, that lung transplants are limited by a person's chances of having a suitable and matching organ donor coupled with lifelong immunosuppressive therapy. About 1000–1500 lung transplants are performed every year in the United States, and they are accompanied by diverse challenges. Recent developments in the field of regenerative medicine and tissue engineering are suggesting possible alternative therapies [230].

### 3.1. Strategies for Pulmonary Regenerative Therapy

The success of lung regenerative medicine will help overcome complications associated with other currently available treatments such as anastomosis failure, material failure, stenosis, and the need for a lifetime of immunosuppression [228]. Pulmonary regeneration is a daunting process, considering its highly specialized cells and complex ECM which shall be subsequently discussed. The regenerative strategies that have been applied to the lungs are broadly categorized as either cellular or a combination of cells and ECM. Although this study is mainly focussed on biomaterials which are essentially the materials that simulate the actual ECM environment, we shall briefly consider the stem cells of the lungs.

### 3.2. Pulmonary Cells with Regenerating Potentials

The lungs, unlike other organs, are comprised of more than 40 different types of highly specialized cells with an equally specialized extracellular matrix; hence, regenerative approaches have been challenging [228]. The lung possesses some intrinsic epithelial regenerative potentials performed by the type II alveolar pneumocytes (epithelial cells) which can proliferate and differentiate into either type I alveolar pneumocytes or more of type II pneumocytes. Other precursor cells in the lungs include the bronchiolar Clara cells and the basal cells of the pseudostratified epithelium of the human airway [228].

Studies have been carried out on the regenerative potential of human airway basal cells [231, 232], where the highly proliferative basal cell population was identified by their expression of KRT5b TP63b [225]. It is also assumed that an additional subset of basal stem cells may exist that include the reported lineage-negative epithelial progenitor (LNEP) cells in the distal part of a healthy lung, which can specifically proliferate after an injury [233]. Schilders et al. characterized basal cells by the expression of Trp63, podoplanin (Pdpn or T1*α*), Ngfr, GSI*β*4, lectin, and cytokeratin 5 (Krt5). They also reported two distinct groups of basal cells: basal stem cells (BSCs) and basal, luminal precursor cells (BLPCs), both of which are also Krt5^+^ and Trp63^+^ [232]. The BSCs asymmetrically divide to give rise to a BSC and one BLPC, which can then differentiate into a secretory cell of a neuroendocrine cell. The BLPCs have a low rate of self-amplification and differentiation, and their expression of Krt8 makes them distinct from BSCs [232]. A small subset of basal cells (<1/5) expressing Krt14 has been shown to have a couple of functions, including maintenance of the Krt5^+^ population of basal cells, regeneration of the ciliated and secretory cells, and rapid upregulation post injury. They may be used as markers for the identification of activated stem cells in the regenerating epithelium [232]. It has been shown that despite sharing similar markers (Trp63^+^/Krt5^+^), the distal alveolar stem cells and the tracheal basal stem cells have different fates in in vivo transplantation and culture. Other multipotent stem cells in the lungs include variant club cells, positive for secretoglobin family 1a member 1 (Scgb1a1) and Cyp2f2-negative. Another subset of Scgb1a1^+^ cells coexpressing the surfactant protein C (Sftpc) are the bronchoalveolar stem cells (BASCs) [232].

### 3.3. The Extracellular Matrix of the Lungs

The lung ECM can authoritatively be called the driver for pulmonary regeneration as it plays diverse functions under the tutelage of cellular behavior, developmental biology, and tissue mechanics [234]. It is the material in the immediate environment of the cells within a tissue, and it actively stimulates the cells. This material includes fibers (such as collagen and reticular and elastic fibers), ground substances (such as glycosaminoglycans, glycoproteins, and proteoglycans), and tissue fluids. The ECM can be said to provide the mechanical and biochemical cues that control fundamental cellular processes such as cell shape and function, cell signaling, cytoskeletal organization and differentiation, changes in proliferation and migration, gene expression and stimulation of polarity, metastatic activity induction, growth factor responses, and formation of stress fibers and focal adhesions, among others [235].

The success or failure of a regenerative construct largely depends on the quality of the underlying extracellular matrix scaffold. The breakdown of the ECM has been reported to contribute to the progression of many lung pathologies [234]. The goal of regenerative medicine, particularly tissue engineering, is to create a natural tissue to replace a damaged body part. Currently, nanotechnology is being used to see how the spatiotemporal profile of the ECM that regulates cellular behaviors can be adequately controlled [236]. Most human cells have their sizes in the microscale range (10–100 *μ*m); however, the ECM that plays the crucial role in almost all cellular functions is in the order of the nanoscales [236]. Recreating the ECM of the lung tissue at the nanoscale has been a daunting task due to the highly complex ECM of the lung tissue, hence the move towards the decellularization of donor human or animal lung tissue as a scaffold and then recellularizing it with the required (stem) cells.

### 3.4. Natural/Biological and Synthetic Biomaterials in Pulmonary Regeneration

Before the whole organ decellularization technique, there has been researching into how the ECM can be reproduced or replicated to support lung regeneration using biomaterials. Biomaterials to be used for lung tissue regeneration must be biocompatible, biodegradable, and porous, and the mechanical integrity should to a large extent be equal that of the native, healthy lung tissue [237].

**Figure 3:**
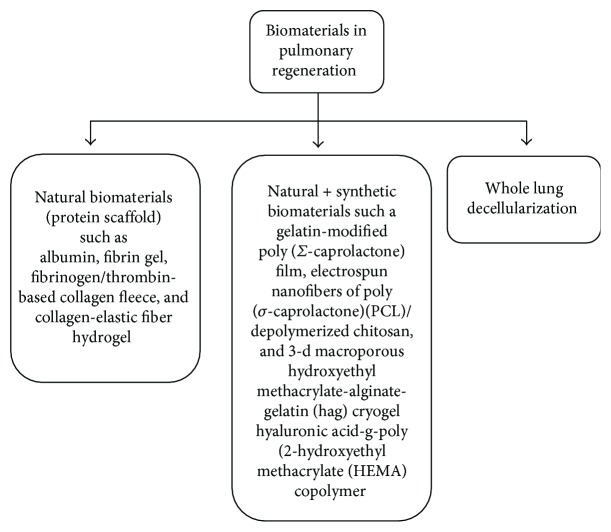
Biomaterials in pulmonary regeneration.

The biomaterials that have been studied in lung regeneration can be broadly categorized as (a) natural or protein-derived or (b) synthetic [238]. Some biomaterials could also consist of both natural and synthetic as shown in this review (Figure 3).

*Natural* scaffolds include collagen, fibrin, hyaluronic acid, glycosaminoglycans, elastin, alginate, chitosan, gelatin, silk, fibronectin, vitronectin laminin, casein, zein, albumin, and growth factors.

*Synthetics* include poly(ε-caprolactone), poly(ethylene glycol), poly(acrylic acid), poly(glycolic acid), poly(lactic acid), poly(lactic-co-glycolic acid), and poly(vinyl alcohol).

Older studies that investigated the use of either biological or synthetic biomaterials for lung tissue regeneration include elastin-based fibrous scaffolds with conducting polyaniline polymers or with a mixture of polyglycolic acid/polylactic acid [238], gel foam sponges [239], and commercial benzyl ester of hyaluronic acid and laboratory cross-linked Hylan [240]. Tracheal scaffolds have also been successfully closely replicated using polyethylene terephthalate (PET) as well as nanocomposites of PET and polyurethane (PU) fibers. However, to reproduce the more complex native lung architecture, moldable synthetic hydrogels like polyvinyl alcohol (PVA), poly(ethylene glycol), and synthetic elastomers [241] like poly(glycerol sebacate) (PGS) have been instrumental [242]. Furthermore, alveolus-like structures have reportedly been formed in collagen hydrogel [243]. Similarly, alveolar-like structures have been produced using a collagen-glycosaminoglycan structure [244]. Polyester biomaterials have also been studied for use in lung regeneration. Lung progenitor cells have also been grown both in vivo and in vitro using polyglycolic acid [245].

These studies have invariably corroborated that a single biomaterial whether biological or synthetic lacks the potential to recapitulate the complex ECM of the lung tissue. This is understandable as the ECM of any tissue is almost never made up of one substance and more so a complex ECM like that of the lungs. Hence, more recent studies have looked into harnessing and combining the individual properties of these biomaterials to create a material that might be similar or able to mimic the actual extracellular matrix of the lungs and also have the ability to facilitate adhesion and support the growth of pulmonary epithelial cells. This review has closely considered the more recent feasible uses of biomaterials for lung tissue regeneration. Hence, this section considers the natural, combined use of natural and synthetic biomaterials, while finalizing with the decellularization technique for lung tissue regeneration.

#### 3.4.1. Natural Biomaterials

*(1) Protein Scaffold: Albumin*. Albumin is the most abundant serum protein in humans and has been shown to influence the attachment of cells to diverse scaffolds like collagen and fibronectin [246]. Albumin can comfortably serve as an interface between cells and scaffolds, hence enhancing the integration of one with the other. Therefore, albumin can be utilized before recellularization of a decellularized lung scaffold to facilitate cellular engraftment to the scaffold. A lot of effort has been garnered into the use of protein as a biomaterial [247, 248] because protein scaffolds like albumin are readily biodegradable and cheap and can be produced in large quantities [249, 250].

The source of albumin is not only the serum but also egg white, milk, and a host of other plant and animal tissues [246]. However, the most commonly used albumin types for tissue engineering scaffold include (a) human serum albumin, (b) porcine serum albumin, and (c) bovine serum albumin [246]. Several techniques can be employed in the synthesis of albumin scaffold; these include freeze-drying methods, chemical/enzymatic cross-linking, solution evaporation, templating and leaching, and 3D printing, among others [251–253]. Overall, for albumin, freeze-drying, cross-linking, heat aggregation, and electrospinning techniques have been shown to be efficient in the production of their scaffolds [246].

Though the application of albumin-based biomaterials has been well established in cardiac, bone, and neural tissue engineering, albumin, to the best of our knowledge, has only been proposed for use as a biomaterial in lung regenerative therapy by Aiyelabegan and colleagues [246]. Therefore, further studies will still be required to establish the use of this material.

*(2) Fibrin Gel*. Angiogenesis plays vital roles in the regenerative alveolarization of adult lungs [254–256]. It has been shown that angiogenic deregulation contributes to the development of chronic lung diseases such as COPD [257], pulmonary fibrosis [258], and bronchopulmonary dysplasia [259, 260]. Hence, understanding the fundamental mechanisms for lung-specific angiogenesis is essential to the development of more efficient methods for lung tissue engineering and regenerative therapy [261]. Polymer fibrils of fibrin gels, produced from thrombin-cleaved fibrinogen, have been shown to trap angiogenic factors such as vascular endothelial growth factor (VEGF) and basic fibroblast growth factor (bFGF) to facilitate angiogenesis in vivo [262]. Subcutaneous implantation of hydrogel has been extensively utilized for research in angiogenesis [263–265]. However, those methods do not summarize organ-specific angiogenesis [261]. Hence, the purpose of one study was to have fibrin gel (hydrogel) implanted directly on the surface of the lung. It was proposed that this system would allow researchers to explore the specific roles that the lung environment plays in angiogenesis and alveolar regeneration [261]. In the study, it was hypothesized that the mechanisms involved in angiogenesis and lung regeneration might have been recapitulated by manipulating the fibrin hydrogel with angiogenic factors similar to the ECM and ECM stiffness [261]. It has indeed been shown that the fibrinogen concentration alters the stiffness of fibrin gel [262]. Thus, manipulating the concentration of fibrinogen may facilitate angiogenesis through either chemical or physical signals [266]. As a result, there is a need to carefully optimize the physicochemical properties of the fibrin gels to recapitulate angiogenesis in an organ-specific manner [261].

*(3) Fibrinogen/Thrombin-Based Collagen Fleece (Tachocombo)*. Besides angiogenic problems associated with chronic lung diseases, we could also have a case of major bleeding from a severe injury of the pulmonary artery. Tachocombo (TC) has previously been used to arrest small bleeding vessels and has been played down with regard to massive vascular injuries [267]. However, Okada et al. demonstrated in their study how TC could be used to secure hemostasis in a large defect created in the pulmonary artery of a canine since the canine's mean pulmonary arterial pressure and wall composition are known to be similar to a human's [268]. The pulmonary artery is a low-pressure apparatus with thin walls, and this makes it a suitable material for injury site compression and TC attachment [267]. TC has been shown to have an advantage over suturing as it prevents vessel stricture [267]. However, it was reported that the most critical aspect of securing hemostasis with TC is the ability to ensure complete adherence and attachment of the TC to the wall of the vessel in a field that is relatively dry [267]. Furthermore, complications associated with such procedures, such as rebleeding, thrombi, stenosis, and pseudoaneurysm, were not observed in the study at 2, 4, and 8 weeks post surgery. Also, the study showed a complete reconstruction of the defect, with minimal scarring, resembling the native vessel at 8 weeks post surgery [267]. However, it appears that this application might be limited to the pulmonary vessels because of the low pressure, as preliminary studies showed rebleeding on the aorta with the same defect size (3 × 3 mm) post TC application [267].

*(4) Collagen-Elastic Fiber Hydrogel*. Collagen is the predominant fiber content of the ECM of human tissue, including lung tissue. Lung parenchymal ECM is mainly composed of collagen types I and III, and these provide the required structural integrity [269]. However, hyaline cartilage support for the lung is essentially collagen type II. The ECM fiber content of the interalveolar septum is mainly reticular collagen and elastic fibers.

Collagen has been utilized in a variety of tissue engineering techniques, but unless further modifications are made, its use is limited to nonload-bearing applications due to its low mechanical properties. Dunphy et al. investigated the effect of adding soluble elastin to collagen hydrogel, and this addition increased the stiffness of the biomaterial [237]. The stiffness of a biomaterial has been demonstrated to influence critical cellular functions such as proliferation and differentiation [270, 271]. The combination of collagen with elastin yielded a biomaterial with high mechanical properties. Also, lung fibroblasts were introduced to the construct and resulted in Young's modulus equaling the theoretical measure of a single alveolar [237]. However, further work will still be required to explore in-depth the viscoelastic properties of this biomaterial in a time-dependent manner, diffusion through the material, and specific lung cell types, and to perform a detailed analysis of the effect of the material on cell phenotypes and behaviors [237].

#### 3.4.2. Combination of Natural and Synthetic

*(1) Gelatin-Modified Poly(ε-caprolactone) Film*. Poly(ε-caprolactone)/PCL has been widely utilized in the field of regenerative medicine and tissue engineering. Kosmala et al. performed some modifications on PCL by immobilizing gelatin on the PCL surface using the amine groups. The modification of PCL increased both the strength and the biocompatibility of the material but also resulted in a loss of flexibility [272]. PCL showed no cytotoxic effects, and the cells spread properly. Furthermore, there was increased cellular proliferation on the modified PCL relative to control [272]. In this study, PCL/gelatin modification did not inhibit the spread of the human epithelial cell line NCI-H292 cells. Also, the cellular metabolic activities increased at 24 hours post seeding to 103% to control [272].

*(2) Electrospun Nanofibers of Poly(ε-caprolactone) (PCL)/Depolymerized Chitosan*. A recent study by Mahoney et al. prepared nanofibers of PCL/chitosan by using water-soluble chitosan. This technique was utilized because acid usually hydrolyzes PCL while it is being prepared for electrospinning and, thus, later weakens the strength of the nanofibers [273]. Trifluoroethanol (TFE), a water-miscible fluorinated alcohol, was used to dissolve PCL, and the TFE also helped to stabilize the PCL/chitosan complexes through hydrogen bonding. Favorable PCL/chitosan molecular interaction is essential to maintaining mechanical and structural integrity for use as a biomaterial in tracheal tissue regeneration therapy. However, owing to its immiscibility, the maximum PCL/chitosan ratio that could be achieved was 70 : 30, and it was virtually impossible to attain greater than 30% chitosan nanofibers [273]. The 80/20 and 70/30 ratios demonstrate minor differences in the nanofibers' morphology concerning cell-to-fiber interaction [273]. The tensile strength of PCL/chitosan has been shown to be much higher than PCL-based composite scaffolds for trachea bioengineering [274]. These scaffolds have also been shown to be nontoxic as the cytotoxic levels of PCL/chitosan nanofibers were shown to be nearly equivalent to PCL and a collagen-coated control [273]. However, additional reinforcing material might be required to achieve a better tensile strength as the PCL/chitosan is expected to degrade and be replaced with regenerated tissue in the long term.

*(3) Hyaluronic Acid-g-Poly(2-hydroxyethyl methacrylate) (HEMA) Copolymer*. Hyaluronic acid (HA) is a component of the ECM which promotes growth as well as the proliferation of cells. Despite its diverse applicability, it has some inherent pitfalls. These include its poor biochemical properties, and its coiled structure gives it an enormous water affinity as it can trap about 1000 times its weight in water, and this ultimately affects its applicability to the field of regenerative medicine [275]. However, several reports have shown that modified HA still appears a suitable material for tissue engineering [276–278]. Radhakumary et al. reported a copolymer of HA and poly(HEMA) [275]. The biomaterial poly(HEMA) is considered one of the most important hydrogels, and its advantages outnumber the other hydrogels [279]. Poly(HEMA) is inert to biological processes, contains water content close to living tissues, is permeable to metabolites, resists degradation, and resists absorption by the body [275]. The copolymer was proven to be an excellent choice for a “natural-synthetic polymer hybrid matrix” and demonstrated the synergistic properties of both materials, such as biocompatibility and water stability [275]. Additionally, in contrast to virgin HA, the copolymer films were found to be stable in water at both neutral and acidic pH. Other advantages also include noncytotoxicity, and most importantly, the copolymer was shown to support multiple cell types, such as alveolar cell adhesion and growth [275].

*(4) 3D Macroporous Hydroxyethyl Methacrylate-Alginate-Gelatin (HAG) Cryogel*. Singh et al. considered a combination of hydroxyethyl methacrylate (HEMA), alginate, and gelatin in lung tissue regeneration [280]. Hydroxyethyl methacrylate (HEMA) is a known highly biocompatible polymer that has been used extensively in tissue engineering [281, 282]. Alginate is also famous for tissue engineering, and it is nontoxic and biodegradable [283, 284]. Gelatin has well-known cell-adhesive properties (arginlyglycylaspartic acid), hence the choice of these three materials [282].

Singh et al. reported that the HAG cryogel does not require surgical intervention to be removed from the system, as the alginate and gelatin components are quickly degraded into smaller fragments that are below the renal threshold [282]. HAG cryogel was shown to be highly elastic and possessed a quick hydration, which indicated that the porous network of polymers is interconnected. It also maintained a good flow rate, and no back pressure was reported [282]. In the study, the implantation of the scaffold was done without cells; however, the scaffold was shown to recruit cells from the surrounding tissue, and the scaffold was completely integrated into the tissue at five weeks. Interestingly, the infiltrated cells were not the expected first-line defense cells, such as the mast cells and dendritic cells that are responsible for graft rejection; hence, biocompatibility of this combination was demonstrated. The HAG cryogel seeded with lung cells showed collagen deposition which is an obligate fiber of the ECM of the lung; as already stated, the cell matrix interaction is an ultimate determinant of the fate of tissue regeneration. However, the study reported a small amount of infiltrating mast cells which were said to have diminished over a few weeks [282].

#### 3.4.3. Whole Lung Decellularization

Decellularization involves the utilization of radiation, detergents, enzymes like nucleases and trypsin, and chemical treatments like acid/alkaline or salt solutions, among others [76]. Although several approaches to decellularization are being used, the optimal decellularization technique is yet to be defined [285]. The breakthrough in organ bioengineering in regenerative medicine where intact lung scaffolding can be decellularized and recellularized is carried out in a bioreactor. The bioreactor is a sterile, closed system with a well-designed mechanism for perfusion and ventilation [286]. The recellularization of the lung scaffold has been performed using the following: autologous, allogeneic, and xenogeneic cell sources; adult, amniotic, differentiated, embryonic, and induced pluripotent cell types; and mixing, perfusion, priming, surface seeding, and microinjection seeding techniques. On the other hand, culture has been done using static approaches such as air exposure, submerging, and, as mentioned earlier, the use of bioreactors [76]. One study proposed a possible alternative approach to deliver cells to the scaffold after implantation, whereby the body's repair mechanism would be harnessed to deliver (stem) cells with their correct spatial organization [73]. This is because during lung injury, fibroblasts and endothelial and endothelial progenitor cells (circulating bone marrow-derived cells) are home to injured sites from the blood. However, Lemon et al. thought it would be speculative to assume this will succeed as it is not clear that the bone marrow contains sufficient amount of precursor cells to facilitate regeneration [73]. However, in a previous study, Lim et al. demonstrated that the decellularized pig's trachea regenerated in vivo without it being recellularized before it was transplanted. They showed the possibility of the body as a bioreactor which, of course, will reduce engineering cost, contamination, and processing time. In the study, they boosted the in vivo regeneration by administering growth factors erythropoietin (EPO) and the granulocyte colony-stimulating factor (GCSF) to enhance the mobilization and differentiation of stem cells and progenitors as an additional therapeutic concept [285]. The limitation, however, was that the mechanisms and pathways were poorly understood and thus further recommendation for a routine clinical application cannot be made.

#### 3.4.4. The Possible Limitations to the Use of Decellularized Scaffold for Regenerative Therapy

**Figure 4:**
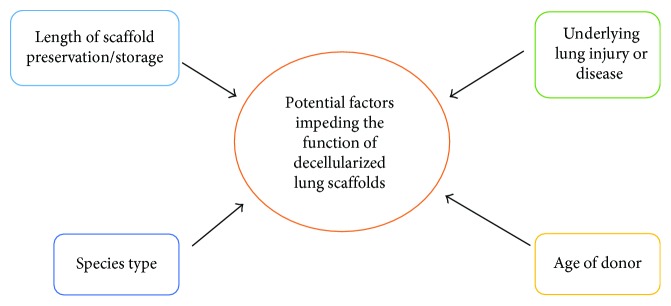
Limitations to the use of decellularized scaffold.

There are a couple of factors that have been reported by literatures to be possible drawbacks to the utilization of some decellularized lung scaffolds as a material for regenerative medicine. Some of these factors may occur in isolation or combination. The factors which may possibly impede the functionality of decellularized lung scaffolds are summarized in Figure 4.

*(1) Age*. The source of the potential donor lung may be an older adult. Thus, research into the effect of age on this ECM biomaterial is necessary to understand its suitability for regenerative therapy. Sokocevic et al. suggested that although organs from a donor of advanced age might appear unsuitable for utilization, the ECM structures required for the initial binding of cells with subsequent growth and proliferation were mostly preserved [73]. Therefore, decellularized aged lungs might be considered for bioengineering approaches, but other varieties of stem cells need to be studied aside from the stromal cell line mesenchymal stem cells (MSCs) used in this study. Furthermore, to the best of our knowledge, no study has been done to determine the age range beyond which a donor's lung becomes unsuitable for bioengineering.

*(2) Underlying Lung Injury or Disease*. The effects of pulmonary diseases like emphysema and fibrosis on the ECM have been reported in some studies. Sokocevic et al. reported that severely diseased lungs would not be considered suitable for decellularization-recellularization, but mild or moderately injured lungs could be regarded as suitable [286]. They showed that despite changes as a result of injury to the lungs (emphysematous changes and fibrosis), ECM structures were appropriately preserved and good initial engraftment was reported. However, despite this initial engraftment, the survival of the MSCs was reduced in the emphysematous lungs. On the other hand, comparable binding, proliferation, and survival were reported in the bleomycin-induced fibrotic lungs, but the more fibrotic zones demonstrated no initial stem cell engraftment and growth [286]. Similarly, another study showed that despite the initial binding of the various stem cells (bronchial epithelial cells, bone marrow-derived MSCs, endothelial cells, and lung fibroblasts) inoculated into decellularized emphysematous lungs, they did not survive for more than one week as opposed to the healthy lungs that survived for about one month [287]. This study also demonstrated that there were no significant differences in the ECM of normal and emphysematous lungs. Wagner et al., in another study, showed the difficulty involved in generating a uniformly decellularized scaffold from a human lung with an underlying interstitial pulmonary fibrosis [287].

*(3) Length of Scaffold Preservation or Storage*. Biological scaffolds, such as those obtained from bone and cartilage, among others, can be stored for a relatively prolonged period before use, especially when treated for instance with low levels of irradiation [288]. However, despite maintaining sterile conditions, it was shown that decellularized lungs should not be stored for more than three months. Also, although the lung scaffold was irradiated at a dose lower than recommended, it showed significant lung ECM distortion and only partly responded to subsequent lung inflation [288]. Baiguera et al. similarly showed that the histoarchitecture of a decellularized trachea became fragmented and less organized after one year, with a relatively loose ECM accompanied by some structural alterations [289]. It was also reported that the angiogenic properties of a one-year decellularized scaffold declined relative to the fresh samples [290]. In this study, they considered a possible restoration of the ECM structure of one-year decellularized tissue by attempting to stabilize the triple-helix structure of the collagen with a natural cross-linking agent known as genipin (1% concentration). This resulted in a more compact, organized matrix that was more resistant to collagenase relative to control and also expressed increased angiogenic potential [291]. However, genipin did not significantly increase the mechanical properties of the one-year stored sample, nor did it affect the organization of its vascular network. This suggests that some long-term ECM deterioration may be irreversible. Genipin has been shown to be a relatively safe cross-linker [292], and a high concentration of genipin might make the scaffold more stable. However, increasing its concentration has reportedly exerted some distinct effects of cell type varieties [293, 294].

*(4) Species Types*. A recent study compared the decellularized scaffolds of different kinds of species: rat, pig, primate, and human. Residual DNA, mechanical property, and fundamental matrix proteins—such as collagen, elastin, and glycosaminoglycans—were assessed in these scaffolds [234]. The study revealed that despite similar levels of collagen among the different species after decellularization, primate and human lung scaffolds were stiffer and possessed more elastin but retained fewer glycosaminoglycans than did rat or pig scaffolds [234]. Moreover, the adhesion of seeded endothelial cells was remarkably enhanced on both the primate and human scaffolds. In all, the decellularized human lung scaffold possessed the ECM profile that closely resembled that of native lung tissue. The primate had modest ECM changes while the rat and pig showed significant losses of ECM [234]. On the other hand, this work was limited by differences in age among the selected species. It can then be inferred from this work that in the search for a scaffold for lung regeneration, the human decellularized scaffold provides an unparalleled ECM. The current biomaterial scaffolds which are used for the lung tissue regeneration are summarized in the Table 3.

## 4. Reflection and Speculations

This review has revealed how complex the cardiopulmonary system is as defined by the individual systemic architectural diversity at the levels of the cells and the extracellular matrix. Hence, the design of any biomaterial for regeneration or therapeutic purposes must be largely based on the natural tissue composition of the damaged site for the efficiency of integration and optimal functionality.

Regeneration of the heart and lungs probably might have been less cumbersome assuming they were an offshoot of the same embryological region. However, the heart is carved out mainly from the lateral mesoderm [113] and the lungs being more of an anterior endodermal offshoot [295], thus accounting for the observed diversity in cellular and tissue composition and thus necessitating research into diverse biomaterials able to efficiently simulate these systems.

Current medical and surgical techniques can only manage cardiopulmonary diseases conservatively and sometimes temporarily but hopefully in the nearest future; regenerative medicine might provide the much needed long-lasting therapy with improvement in the quality of life.

Understanding that the histology of the lungs vastly varies from the conducting zone to the respiratory zone, which also largely differs from that of the heart, should direct the therapeutic approach to be employed especially when biomaterials have to be utilized. The biomaterials have to be tailored to suit the damaged tissue area for it to be properly integrated into the host tissue. However, this is currently a daunting task.

Interestingly in 2013, Peng et al. made an important discovery describing the codevelopment of the cardiopulmonary system. The team demonstrated multipotent population of cardiopulmonary mesodermal progenitors (CPPs) at the posterior cardiac pole expressing Wnt2, Isll, and Glil. Regulating the CPPs was the sonic hedgehog (Shh) expressed from the foregut endodermal origin of the primitive tracheal diverticulum (lung bud). This facilitates the connection of the heart to the pulmonary vasculature [295]. These CPPs produce the mesodermal lines of the cardiac inflow tract and lungs, also including the cardiac cells, pulmonary vascular and smooth muscles of the airways, proximal vascular epithelial cell, and cells resembling pericytes.

The excitement here, although still premature, is the possibility of having some parts of the cardiopulmonary system especially the pulmonary vascular tree being regenerated in concert with the mesodermal derivatives of the lungs. Probably, these multipotent progenitor cells in combination with other progenitors of epithelial and endothelial cells, incorporated with the right signals or growth factors, and transplanted on the right biomaterial platform might just make the right concoction.

In speculating for a pathology like COPD, especially chronic bronchitis, there is irreversible damage to the wall of the organ. These CPPs, if translated with the right biomaterial, might just help in the regeneration of the mesodermal derivatives. However, this might not apply to emphysema, another COPD, which is largely a problem with elastic fibers at the distal alveoli as the CPPs do not extend this far embryologically [289]. However, stem cell therapy is currently not advocated for COPDs (https://www.copdfoundation.org).

Another speculation might be for the decellularized scaffolds for which a couple of negative factors have been identified. Decellularization takes out not only the epithelial cells but also cells of mesodermal origin. Hence, these scaffolds might benefit from incubation in these CPPs alongside other cells as they might help with regenerating the core of the airway such as the cartilages, smooth muscles, connective tissue, and blood vessels, among others.

Although this discovery and its potential therapeutic applications are still in its infancy, it certainly has helped to demystify the gray area in cardiopulmonary embryologic development.

Hence, the CPP-biomaterial combination might be an area for future research considerations for cardiopulmonary regeneration and therapies.

## 5. Conclusion

Several therapies have been trialed in the treatment of cardiopulmonary diseases, but they have yet to provide a desired quality of life. Likewise, organ transplants are not a readily available therapy and, when available, only provide a temporary palliation as they are not devoid of complications. These issues have fueled the exploration of stem cells and regenerative medicine to find ways to repair damaged cardiac tissue, using not only stem cells but biomaterials that could replace the damaged environment in which the cells reside. This review has shown that it will probably become a necessity to combine biomaterials, either biological or synthetic, to stimulate the damaged ECM while, equally, incorporating more recent nanotechnology techniques. However, owing to the complexities involved in recapitulating this ECM, decellularizing and recellularizing of donor tissues or organs appear to be a reprieve, but this also is not free of its challenges. Thus, further research is still required to explore the synergy of biomaterials and improve decellularization-recellularization methods. This review also speculated on the possible regenerative potentials of CPPs which is currently at its infancy.

## Figures and Tables

**Table 1:** Classifications of natural and synthetic biomaterials used in cardiac regeneration.

Biomaterials		Experimental studies			References
Classification	Subclassification	In vitro	In vivo	Clinical trials	
Natural biomaterials: polysaccharide-derived	Chitosan	Yang et al. (2009): chitosan improved silk fibroin effect on rat MSC. Liu et al. (2013): chitosan improved the differentiation of ADSC.	Chi et al. (2013): BASC on chitosan improved overall cardiac function in MI rat models.	None reported.	[29, 33, 39, 40]
			Wang et al. (2010): chitosan improved the function of bFGF on cardiac function.		
	Alginate	Wang et al. (2012): hydrogels from alginate can enhance the growth of stem cells.	Leor et al. (2000): RFCC in alginate scaffolds supported neovascularization in rat models.	None reported.	[42, 47, 48]
			Yeghiazarians et al. (2012): hESC with inhibited p38 mitogen-activated protein kinase on alginate scaffolds improved cardiac function with no immune response.		
	Agarose	Dahlmann et al. (2013): agarose microwells supported the differentiation of pluripotent stem cells to cardiomyocytes.		None reported.	[58]
	Hyaluronic acid	Yang et al. (2010): HA combined with SF seeded with rat MSCs enhanced cardiac gene expression.	Yoon et al. (2016): HA modified with polyethylene glycol-thiol reduced infarct size and promoted neovascularization in a rat model.	None reported.	[64–66]
		Göv et al. (2016): HA and gelatin enhanced the differentiation of human ADSC to CM.			

Natural biomaterials: protein-derived	Collagen	Yu et al. (2017): type I collagen with carbon nanotubes boosted cardiac cell function.	Frederick et al. (2010): collagen-gold nanocomposite coated with MSCs improved neovascularization.	None reported.	[79–82]
		Sun et al. (2017): collagen hydrogels and carbon nanotubes improved cell alignment.	Hsieh et al. (2016): vitronectin-collagen improved ventricular function in rat models.		
	Fibrin	Ye et al. (2013): fibrin scaffolds with thymosin *β*4 sustained swine MSC.	Ichihara et al. (2017): epicardial placement of bone marrow MSC in fibrin scaffold should have better retention of the MSC.	Menasché et al. (2014): trials in observing the prospects of fibrin patch with hESC-CPC on individuals with heart failure. To be completed in 2018	[87–90, 93]
		Nie et al. (2010) and Yang et al. (2012): fibrin scaffold manipulated by growth factors resembled native ECM of the human heart.			
	Gelatin	Navaei et al. (2016): Ultraviolet cross-linkable gold nanorod-incorporated gelatin ethacrylate hybrid hydrogels improved cell metabolic activity.	Takehara et al. (2008): gelatin scaffold + bFGF + human cardiosphere-derived cells had a higher ejection fraction in pig MI models.	Yacoub et al. (2013) illustrated that bFGF in biodegradable gelatin hydrogel sheet implanted on the epicardium of human patients with ischemic cardiomyopathy and heart failure leads to the continuous release of bFGF.	[98–100]
	Matrigel	Lam et al. (2017): matrigel enhanced the type I collagen matrix.	Zhang et al. (2017): matrigel and endothelial stem cells improved vascularization and electrical activity.	None reported.	[105, 106]
	Cardiogel	Chang et al. (2007): MSCs on cardiogel had better cellular expansion.	Matsuda et al. (2013): ASCs on cardiogel supported angiogenesis.	None reported.	[112, 115]
	Decellularized extracellular matrix	Pagano et al. (2017): CPCs thrived on healthy DECM.	Söylen et al. (2017): nonseeded decellularized homografts from human donors reduced complications with bovine jugular vein conduits.		[125, 129, 132]
		Lee et al. (2015): DECM from rat preserved and improved the survival of CM.			

Synthetic		Mukherjee et al. (2011): poly(*ε*-caprolactone) combined with poly(L-lactic acid) and collagen supported rabbit CM.	Sugiura et al (2016): poly(L-lactic-co-*ε*-caprolactone) and polyglycolic acid supported human-induced pluripotent stem cell-derived CM in athymic rat.	None reported.	[145, 147, 148]
		Castilho et al. (2017): poly(hydroxymethyl glycolide-co-*ε*-caprolactone) with melt electrospinning writing aligned the growth of cardiac progenitor cells.	Somasuntharam et al. (2013): polyketals serve as good vehicles for delivering siRNA to the MI heart.		

**Table 2:** The current applications of nanotechnology and tissue engineering in cardiac regeneration.

Biomaterials		Experimental studies			References
Classification	Subclassification	In vitro	In vivo	Clinical trials	
Nanotechnology	Cardiac patch production	Yamato and Okano (2004): grafting poly(*N*-isopropylacrylamide) to a culture surface that is thermoresponsive in order to produce scaffold-free cardiac tissue.	Jang et al. (2017): 3D-printed prevascularized stem cell patch with CSC and MSC improved cardiomyogenesis and neovascularization.	None reported.	[172, 175–177]
		Fleischer et al. (2014): poly(*ε*-caprolactone), dichloromethane, and dimethylformamide which was electrospun resembled perimysial fibers.	Gaebel et al. (2011): PEUU seeded with hMSC and HUVEC enhanced capillary density.		
	Improving drug and therapy delivery systems	Cheng et al. (2016): 5-azacytidine delivered by FMNSs induced the differentiation of P19 cells to CM.	Change et al. (2013): the PLGA-IGF-1 NP complex showed increased IGF-1 retention, induced the phosphorylation of Akt, and provided early cardioprotection postmyocardial infarction.	None reported.	[189, 198–200, 212]
		Oh et al. (2006); Lee and Yuk (2007): a pluronic-based micelle and liposomal system was developed and designed as a core/shell NP with a lecithin core loaded with a growth factor and a pluronic shell and showed prospect in drug delivery.	Pascual-Gil et al. (2015): intramyocardial injection of NP-liraglutide in a rat model of myocardial infarction sufficiently improved cardiac function.		
	Nanoparticles and magnets: role in stem cell retention	None reported.	Vadergriff et al. (2014): ferumoxytol NPs in the presence of heparin and protamine were used to label stem cells.	None reported.	[215, 216]
			Zhang et al. (2012): MNBs/AdhVEGF complex showed significant improvement in left ventricular function.		
	Differentiation of endometrial stem cells	Barabadi et al. (2016) showed that endometrial stem cells can be conveniently programmed into CM.	None reported.	None reported.	[217, 219]
	Loss-of-function studies	None reported.	Diao et al. (2015): on a zebrafish model, retinoic acid signaling was compromised by siAldh1a2 therapy.	None reported.	[220]

Tissue engineering		Sidorov et al. (2017): identified the potentials of the “I-wire” platform in controlling the applied force on ECTCs while cross-examining their inactive and active mechanical and electrical characteristics.	Emmert et al. (2017): rapid fabrication of fibrous, semilunar heart valve scaffolds for the ovine model.		[223, 226]

**Table 3:** Classifications of biomaterials used in lung tissue regeneration.

Biomaterials		Experimental studies			References
Classification	Subclassification	In vitro	In vivo	Clinical trials	
Natural biomaterials	Albumin	Aiyelabegan et al. (2016): albumin enhanced the integration of cells and scaffolds with one another.	None reported.	None reported.	[246]
	Fibrin gel	None reported.	Mammoto et al. (2013): polymer fibrils of fibrin gels trapped VEGF and bFGF and enhanced angiogenesis in a rat model	None reported.	[260]
	Fibrinogen/thrombin-based collagen fleece	None reported.	Ikeda et al. (2011): TC is better than suturing because it prevents vessel stricture in a canine model.	None reported.	[267]
	Collagen-elastic fiber hydrogel	Hadjipanayi et al. (2009): influenced cellular proliferation and differentiation	None reported.	None reported.	[270]

Combination of natural and synthetic biomaterials	Gelatin-modified poly(*ε*-caprolactone) film	Kosmala et al. (2016): PCL/gelatin modification did not stop human epithelial cell line NCI-H292 cells to proliferate.	None reported.	None reported.	[272]
	Electrospun nanofibers of poly(*ε*-caprolactone)(pcl)/depolymerized chitosan	Mahoney et al. (2016): PCL/chitosan molecular interaction helped maintain the architecture of tracheal tissue regeneration therapy.	None reported.	None reported.	[273]
	Hyaluronic acid-g-poly (2-hydroxyethyl methacrylate (hema) copolymer	Radhakumary et al. (2011): copolymer of HA and poly(HEMA) was observed as the best choice for the “natural-synthetic polymer hybrid matrix”	None reported.	None reported.	[275]
	3D macroporous hydroxyethyl methacrylate-alginate-gelatin (hag) cryogel	Singh et al. (2011): combining HEMA, alginate, and gelatin improved lung tissue regeneration.	None reported.	None reported.	[280]
